# Elevated fibrinogen-albumin ratio is an adverse prognostic factor for patients with primarily resected gastroesophageal adenocarcinoma

**DOI:** 10.1007/s00432-024-05976-z

**Published:** 2024-10-14

**Authors:** Gerd Jomrich, Winny Yan, Dagmar Kollmann, Ivan Kristo, Daniel Winkler, Hannah Puhr, Aysegül lhan-Mutlu, Marlene Hollenstein, Reza Asari, Sebastian F. Schoppmann

**Affiliations:** 1 Department of General Surgery, Gastroesophageal Tumor Unit, Comprehensive Cancer Center (CCC), Medical University of Vienna, Vienna, Austria; 2 Vienna University of Economics and Business, Vienna, Austria; 3 Department of Medicine 1, Division of Oncology, Gastroesophageal Tumor Unit, Medical University of Vienna, Comprehensive Cancer Center (CCC), Vienna, Austria; 4 Department of Laboratory Medicine, Medical University of Vienna, Vienna, Austria

**Keywords:** Esophageal cancer, Fibrinogen to albumin ratio, Prognostic parameter, Malignant tumor, Prognosis

## Abstract

**Purpose:**

Serum fibrinogen and albumin play important roles in systemic inflammation and are implicated in tumor progression. The fibrinogen-to-albumin ratio (FAR) has shown a prognostic impact in several malignancies. This study aims to assess the prognostic value of the pretherapeutic FAR in patients with adenocarcinoma of the gastroesophageal junction (AEG) who underwent upfront resection.

**Methods:**

Consecutive patients who underwent surgical resection at the Department of Surgery at the Medical University of Vienna between 1992 and 2014 were included into this study. Optimal cut-off values were determined with the receiver-operating characteristic (ROC) curve, uni- and multivariate analyzes were calculated by the Cox proportional hazard regression model for overall survival (OS).

**Results:**

Among 135 included patients, the majority were male (79.26%), with a mean age of 66.53 years. Elevated FAR correlated significantly (*p* = 0.002) with shorter OS in univariate analysis, also confirmed as independent prognostic factor (*p* = 0.005) in multivariable analysis. The ROC curve of FAR (AUC = 0.744) outperformed fibrinogen (AUC = 0.738) and albumin (AUC = 0.378) in predicting OS for AEG patients.

**Conclusion:**

The FAR serves as an independent prognostic factor for OS in patients undergoing primarily resection for AEG. Given its routine availability and ease of calculation, FAR could help in diagnosis and treatment selection for AEG patients. Further validation studies are warranted to confirm these findings conclusively.

**Supplementary Information:**

The online version contains supplementary material available at 10.1007/s00432-024-05976-z.

## Introduction

According to the latest GLOBOCAN analysis, esophageal cancer (EC) is the seventh most common type of cancer worldwide (Sung et al. 2021). Two histological subtypes are distinguishable, differing geographically, genetically, and in risk factors: esophageal squamous cell carcinoma (ESCC) and adenocarcinoma of the gastroesophageal junction (AEG). ESCC generally prevails, however, recent decades have seen a notable rise in AEG diagnoses, especially in western nations, surpassing ESCC (Arnold et al. 2015). Although survival rates for AEG have improved with multimodal therapies, the prognosis remains poor with a maximum 5-year survival rate of 20% in high-income countries (Morgan et al. 2020). Despite improvements in the treatment of AEG combining surgical resection and new systemic therapy regimens, approximately 50% of patients experience disease recurrence (Dhakras et al. 2020).

Currently, conventional tumor-based histopathologic risk factors, including tumor and lymph node staging, tumor differentiation, and resection margin status, are the sole prognostic indicators only accessible post-surgery. Therefore, identifying prognostic markers before surgery can enhance treatment approaches for patients with EAG (Dhakras et al. 2020).

Over the past decade, numerous biomarkers have emerged to predict patients survival rates in various malignancies (An et al. 2022; Jomrich et al. 2019, 2020, 2021; Tada et al., 2023). Among these parameters aiding in individual risk assessment in EC is the FAR, which is calculated using preoperative available laboratory tests reflecting the influence of immune response on tumor progression and survival (Diakos et al. 2014; Dinca et al. 2023). Elevated FAR is associated with decreased OS across various cancers including esophageal squamous cell carcinoma and gastric carcinoma, confirming its potential as a prognostic marker for patients suffering from malignant diseases (Zhang and Xiao 2019). Additionally, inflammatory parameters are linked to decreased disease-free survival (DFS) and OS in patients with AEG (Powell et al., 2021).

Fibrinogen is an acute-phase protein, which acts as a key role in the coagulation cascade and hemostasis. It has been ascribed an important role in the stabilization of tumor cells, their cell invasion, the metastasis process and the promotion of blood vessel formation in malignant lesions (Palumbo et al. 2000). The correlation between plasma fibrinogen levels and prognosis has been observed in several types of cancers including AEG (Jagadesham et al. 2017).

Serum-albumin is being used to determine the nutritional status since malnutrition and chronic systemic inflammation in cancer affect albumin synthesis (Simons et al. 1999). Cytokines like interleukin-6 or tumor necrosis factor, released during the systemic inflammatory response, stimulate acute-phase protein production such as fibrinogen. This increases amino acid demand and alters vascular wall permeability, enhancing albumin uptake into cells (Barber et al. 1999). Serum albumin has been identified as an independent predictor of survival in different cancer types (Maltoni and Amadori 2002), including lung (Yuan et al., 2022), gastric (Oñate-Ocaña et al. 2007) and pancreatic (Siddiqui et al. 2007) cancer. Thus, pretreatment serum albumin levels offer valuable prognostic significance in cancer, showing the less the albumin value, the worse the prognosis (Gupta and Lis 2010).

The aim of this study was to assess whether the FAR, obtained preoperatively from patients with AEG, can predict survival rates. While it has demonstrated a prognostic value in ESCC (Tan et al. 2017), significance of FAR for AEG remains uncertain, driving the focus of this study. To the best of our knowledge, this is the first study investigating the prognostic role of FAR in primarily resected AEG.

## Methods

This retrospective study uses data from a prospectively maintained database from the Department of General Surgery at the Medical University of Vienna, of patients who underwent primarily resection for AEG between 1992 and 2014. The classification of the primary tumor was based on the eighth edition of the American Joint Committee on Cancer (AJCC) (Rice et al. 2017). Pre- and post-operatively every patient was discussed in the interdisciplinary tumor board meeting. Patients receiving adjuvant therapy were treated according to the standards of the Comprehensive Cancer Center of the Medical University of Vienna at the time of presentation, with cisplatin/5-fluorouracil based regimens. The location of tumors at the gastroesophageal junction was classified according to Siewert and Stein (Siewert and Stein 1998). The surgical procedure was chosen depending on primary tumor location (abdominothoracic en-bloc esophagectomy or trans-hiatal extended gastrectomy, respectively).

Inclusion criteria comprised availability of serum fibrinogen and albumin laboratory values obtained within two weeks before surgery, histologically confirmed diagnosis of AEG, and absence of neoadjuvant therapy. Exclusion criteria included pregnancy, age under 18 years at disease onset. Patients with fever or signs of active or chronic inflammatory disease during laboratory data collection were excluded as well. Serum fibrinogen and albumin were analyzed in the central laboratory of the Vienna General Hospital, which has a certified (ISO 9001) and accredited (ISO 15189 since 2008) quality management system.

The baseline clinicopathological values were retrospectively reviewed and collected from the local database and electronical patients records. The patients’ baseline clinicopathologic values were retrospectively reviewed and collected from the local database and electronic patient records. Follow-up appointments were scheduled every three months for the first two years and every six months for the subsequent three years.

### Statistical analysis

The analysis and correlation of the data used was carried out using IBM SPSS Statistics 29.0 (Armonk, New York, USA) software for Windows. A significance threshold of *p* < 0.05 was applied for statistical significance. Overall survival (OS) was defined as the time between primary surgery and the patient’s death by any cause. Categorical variables were described by absolute and relative frequencies, and metric variables were described by mean and median minimum and maximum.

The FAR was defined as the fibrinogen levels divided by the serum albumin levels. Receiver-operating characteristic (ROC) curve analysis was used to determine the optimal cut-off values for fibrinogen, albumin, and FAR. The determination of these cut-off values was considered by maximizing Youden’s index (sensitivity + specificity − 1). Univariate and multivariate analyses were conducted to assess the prognostic value of FAR. In the multivariate analysis, each parameter - FAR, albumin, and fibrinogen - was individually evaluated using the Cox regression proportional hazard model to ascertain their independent prognostic significance for OS. Furthermore, separate multivariate models, using clinical or pathological staging were performed.

The association between FAR and clinicopathologic factors was evaluated through the Chi-square and Kruskal-Wallis tests. Survival analysis, including OS and survival curves, was performed using the Kaplan-Meier survival curves. The disparity in survival outcomes between various groups was determined using the log-rank test. Given the exploratory nature of the study, no adjustments for multiple testing were made unless explicitly stated (Bender and Lange 2001).

## Results

**Fig. 1:**
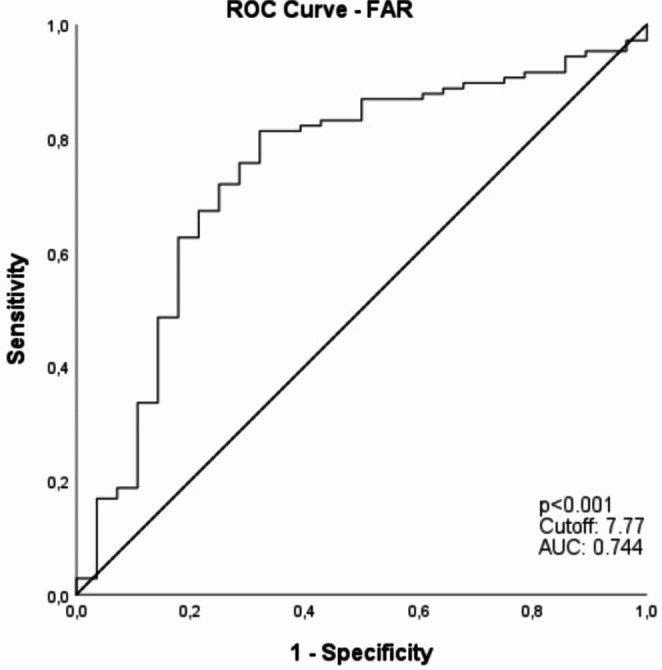
ROC curve analysis of serum FAR

**Fig. 2:**
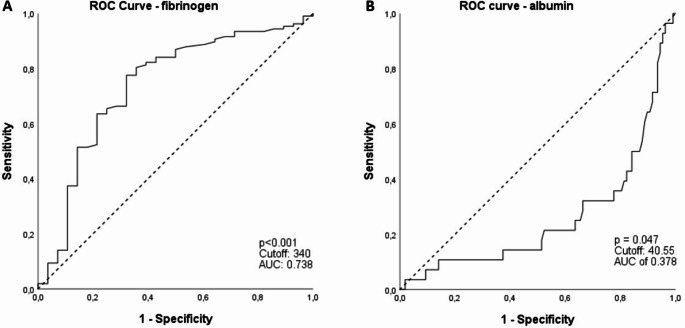
ROC curve analysis. **(a)** of serum albumin and **(b)** of serum fibrinogen

In the present study, a total of 135 patients (107, 79.26% male) were included (suppl. Figure 1). Mean age at the time of surgery was 66.53 years (range 44–90 years), 81 patients (60%) were 65 years old or older on the day of surgery. The correlation between the clinicopathological parameters and FAR are compiled in Table 1. The OS was settled as the endpoint, using the ROC analysis to determine the cut-off value of FAR. The Youden index calculated revealed an optimal cut-off for FAR = 7.77 (Fig. 1), fibrinogen = 340 (Fig. 2a) and albumin = 40.55 (Fig. 2b), respectively.

**Table 1:** Association of the FAR with clinicopathologic parameters

Variable		High FAR (*n* = 97)	(%)	Low FAR (*n* = 38)	(%)	*p*-value
mean Age (SD)		67.20 (10.92)		64.84 (8.49)		0.053
Age						0.061
	Age ≥ 65	63	(64.9%)	18	(47.4%)	
	Age < 65	34	(35.1%)	20	(52.6%)	
Sex						**0.021**
	Male	72	(74.2%)	35	(92.1%)	
	Female	25	(25.8%)	3	(7.9%)	
G						0.571
	1	4	(4.1%)	1	(2.6%)	
	2	49	(50.5%)	16	(42.1%)	
	3	44	(45.4%)	21	(55.3%)	
cT						0.199
	1	23	(23.7%)	14	(37%)	
	2	41	(42.3%)	16	(42%)	
	3	33	(34%)	8	(21%)	
cN						**0.026**
	0	25	(25.8%)	20	(52.6%)	
	1	62	(63.9%)	16	(42.1%)	
	2	8	(8.3%)	2	(5.3%)	
	3	2	(2.1%)	0	(0%)	
pT						**0.022**
	1	24	(24.7%)	19	(50%)	
	2	33	(34.02%)	12	(31.6%)	
	3	36	(37.1%)	6	(15.8%)	
	4	4	(4.1%)	1	(2.6%)	
pN						0.120
	0	40	(41.2%)	23	(60.1%)	
	1	34	(35%)	12	(31.6%)	
	2	14	(14.4%)	2	(5.3%)	
	3	9	(9.3%)	1	(2.6%)	
Tumor type						0.208
	AEG I	50	(51.6%)	19	(50%)	
	AEG II	44	(45.4%)	15	(39.5%)	
	AEG III	3	(3%)	4	(10.5%)	
Surgery technique						0.995
	Single cavity	46	(74.4%)	18	(47.4%)	
	Two-cavity	51	(52.6%)	20	(52.6%)	
Adjuvant therapy						**0.019**
	Yes	55	(56.7%)	13	(34.2%)	
	No	42	(43.3%)	25	(65.8%)	
ASA						0.438
	I	10	(10.3%)	7	(18.4%)	
	II	73	(75.3%)	28	(73.7%)	
	III	12	(12.4%)	3	(7.9%)	
	IV	2	(2.1%)	0	(0%)	
ECOG						0.828
	0	36	(37.1%)	14	(36.8%)	
	1	45	(46.4%)	19	(50%)	
	2	14	(14.4%)	5	(13.2%)	
	3	2	(2.1%)	0	(0%)	

Abbreviations: *SD* standard deviation, *c* clinical staging, *p* pathological staging, *ASA* American society of anesthesiologists classification, *AEG* Adenocarcinoma of the gastroesophageal junction *ECOG* Eastern Cooperative Oncology Group Performance Status, *HR* hazard ratio, *CI* confidential interval;

**Fig. 3:**
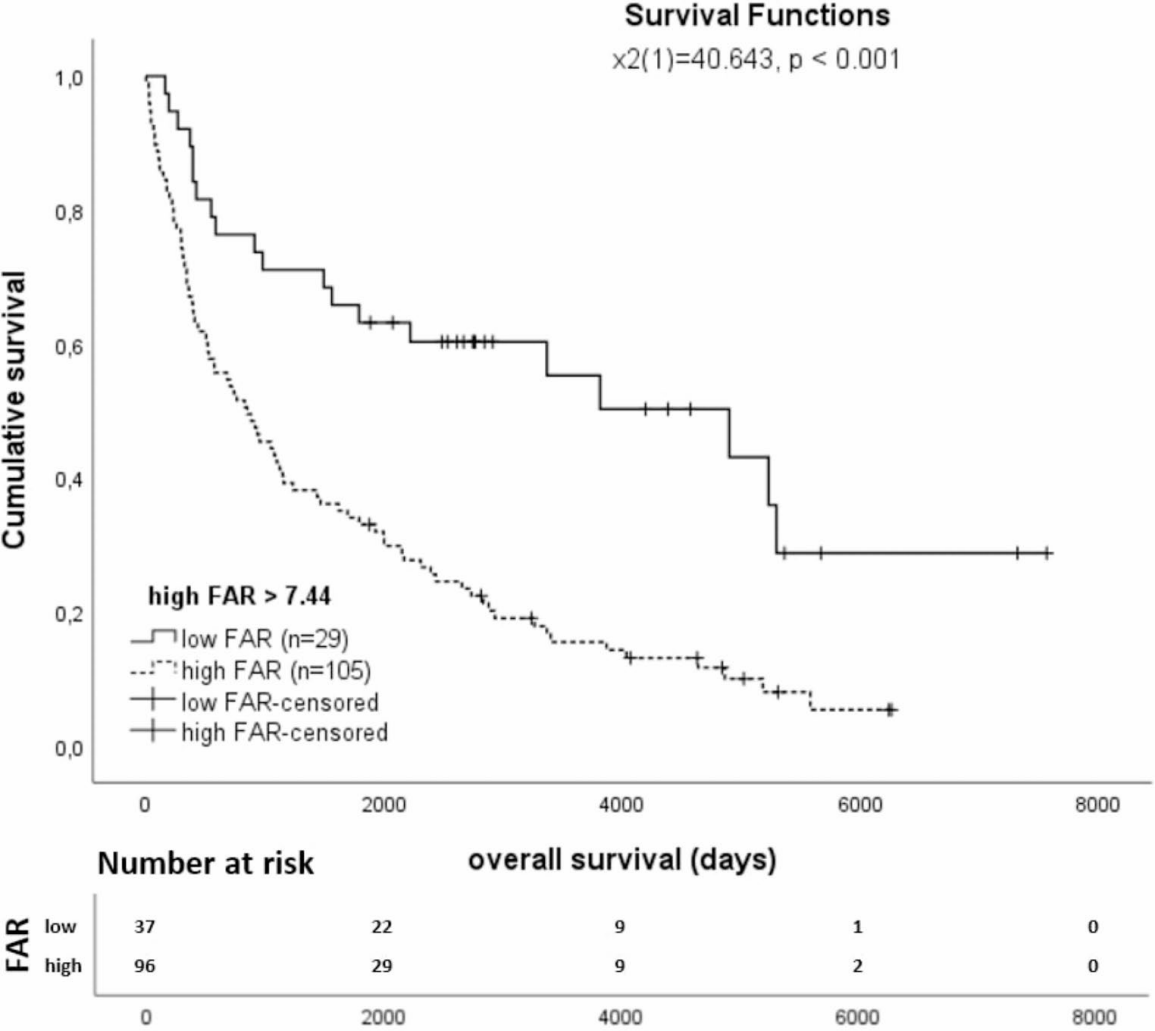
Kaplan-Meier survival curves stratified by serum FAR in primarily resected AEG patients

For all patients the median survival of all patients was 37.4 months, and the Kaplan-Meier analysis shows that the group with high FAR has a significantly (*p* < 0.001) worse outcome compared to the group with low FAR (Fig. 3).

The univariate analysis shows significant correlation of OS and preoperative UICC stage (*p* < 0.001), clinical tumor stage (*p* < 0.001), clinical lymph node stage (*p* < 0.001), postoperative UICC stage (*p* < 0.001), pathologic tumor stage (*p* < 0.001), pathologic lymph node stage (*p* < 0.001), tumor grading (*p* = 0.002), adjuvant therapy (*p* < 0.001), fibrinogen (*p* = 0.013), and FAR (*p* = 0.002).

Multivariate analysis was conducted using different models: one incorporating clinical staging, and another one incorporating pathological staging. Furthermore, separate models for FAR, fibrinogen and albumin were calculated to avoid redundancy or interaction in between these factors. In the multivariate Cox regression model with clinical staging (cT, cN) (Table 2), FAR was an independent predictor of OS with a hazard ratio (HR) of 2.456 (*p* < 0.001), indicating a 145.6% higher risk of earlier death. The clinical tumor stage (cT) was also significant (*p* = 0.005), particularly for cT2 with an HR of 2.768 (*p* = 0.001), indicating a 176.8% higher risk of earlier death. Clinical lymph node involvement (cN) was not significant overall (*p* = 0.246), although cN2 approached significance (HR = 2.489, *p* = 0.051).

**Table 2:** Clinical staging model - univariate and multivariate Cox regression analyses estimating the influence of the FAR and clinicopathologic parameters on OS in patients with primarily resected AEG

Variable	Univariate			Multivariate		
	HR	95% CI	*p*-Value	HR	95% CI	*p*-Value
Age (years)	1.013	0.992–1.034	0.222			NI
Age65 (ref. ≥ 65)	1.243	0.838–1.843	0.280	1.051	0.687–1.606	0.819
SEX (ref. male)	1.202	0.757–1.909	0.437	0.732	0.441–1.215	0.228
G			**0.002**			0.018
1 vs. 3	0.421	0.151–1.174	**0.098**	1.658	0.572–4.806	0.352
2 vs. 3	0.501	0.336–0.747	**< 0.001**	3.133	1.021–9.617	**0.046**
cT			**< 0.001**			**0.006**
1 vs. 3	1.635	0.973–2.748	0.064	1.639	0.944–2.845	**0.079**
2 vs. 3	3.835	2.238–6.570	< 0.001	2.751	1.478–5.123	**0.001**
cN			**< 0.001**			0.259
1 vs. 0	2.025	2.025–3.143	**0.002**	1.418	0.869–2.315	0.163
2 vs. 0	6.380	3.001–13.564	**< 0.001**	2.457	0.977–6.181	0.056
3 vs. 0	2.636	0.625–11.115	0.187	1.126	0.250–5.082	0.877
pT			**< 0.001**			NI
1 vs. 4	0.099	0.036–0.272	**< 0.001**			
2 vs. 4	0.325	0.125–0.843	**0.021**			
3 vs. 4	0.595	0.232–1.523	0.279			
pN			**< 0.001**			NI
1 vs. 0	0.157	0.075–0.328	**< 0.001**			
2 vs. 0	0.517	0.257–1.040	0.064			
3 vs. 0	0.998	0.451–2.208	0.996			
Adjuvant therapy (ref. yes)	0.239	0.157–0.364	**< 0.001**			NI
ASA			0.507			0.566
I	1.088	0.610–1.940	0.776	0.894	0.458–1.746	0.743
II	1.651	0.765–5.651	0.202	0.585	0.219–1.565	0.286
III	0.739	0.097–5.651	0.770	0.293	0.031–2.758	0.284
ECOG			**< 0.001**			**0.009**
ECOG 1	0.950	0.627–1.438	0.808	0.848	0.542–1.327	0.471
ECOG 2	1.063	0.578–1.956	0.844	0.905	0.425–1.928	0.796
ECOG 3	39.862	7.050-225.383	**< 0.001**	20.414	2.897-143.837	**0.002**
Fibrinogen	1.002	1.000-1.004	**0.013**			*See suppl. Table * 1
Albumin	0.969	0.939-1.000	0.053			*See suppl. Table * 2
FAR	0.1082	1.030–1.136	**0.002**	2.433	1.419–4.174	**0.001**

Abbreviations: *NI* not included, *c* clinical staging, *p* pathological staging, *OP* operation, *HR* hazard ratio, *CI* confidence interval, *ASA* American society of anesthesiologists classification, *ECOG* Eastern Cooperative Oncology Group

Bold values indicate statistical significance

In the pathological staging model (pT, pN) (Table 3), FAR remained a significant predictor with an HR of 2.170 (*p* = 0.004), indicating a 117% higher risk of earlier death. The pathological tumor stage (pT) was highly significant overall (*p* < 0.001), with pT 1 (HR = 3.434, *p* < 0.001), pT 2 (HR = 4.289, *p* < 0.001), and pT3 (HR = 4.512, *p* = 0.014) all showing substantial increased risks of earlier death. Pathological lymph node involvement (pN) was not significant overall (*p* = 0.056), but pN1 (HR = 1.889, *p* = 0.029) and pN2 (HR = 2.701, *p* = 0.011) were significant.

**Table 3:** Pathological staging model - univariate and multivariate Cox regression analyses estimating the FAR and clinicopathologic parameters on OS in patients with primarily resected AEG

Variable	Univariate			Multivariate		
	HR	95% CI	*p*-Value	HR	95% CI	p-Value
Age (years)	1.013	0.992–1.034	0.222			NI
Age65 (ref. ≥ 65)	1.243	0.838–1.843	0.280	1.216	0.780–1.897	0.387
SEX	1.202	0.757–1.909	0.437	0.749	0.442–1.269	0.283
G			**0.002**			0.148
1 vs. 3	0.421	0.151–1.174	0.098	1.366	0.472–3.951	0.565
2 vs. 3	0.501	0.336–0.747	**< 0.001**	2.117	0.702–6.386	0.183
cT			**< 0.001**			NI
1 vs. 3	1.635	0.973–2.748	0.064			
2 vs. 3	3.835	2.238–6.570	< 0.001			
cN			**< 0.001**			NI
1 vs. 0	2.025	2.025–3.143	**0.002**			
2 vs. 0	6.380	3.001–13.564	**< 0.001**			
3 vs. 0	2.636	0.625–11.115	0.187			
pT			**< 0.001**			**0.001**
1 vs.4	0.099	0.036–0.272	**< 0.001**	3.402	1.752–6.608	**< 0.001**
2 vs. 4	0.325	0.125–0.843	**0.021**	4.377	2.109–9.084	**< 0.001**
3 vs. 4	0.595	0.232–1.523	0.279	5.008	1.462–17.154	**0.010**
pN			**< 0.001**			**0.065**
1 vs. 0	0.157	0.075–0.328	**< 0.001**	1.925	1.085–3.414	**0.025**
2 vs. 0	0.517	0.257–1.040	0.064	2.554	1.174–5.554	**0.018**
3 vs. 0	0.998	0.451–2.208	0.996	2.269	0.940–5.480	**0.069**
Adjuvant therapy (ref. yes)	0.239	0.157–0.364	**< 0.001**			NI
ASA			0.507			0.167
I	1.088	0.610–1.940	0.776	0.460	0.222–0.953	**0.037**
II	1.651	0.765–5.651	0.202	0.359	0.122–1.058	**0.063**
III	0.739	0.097–5.651	0.770	0.459	0.047–4.487	0.503
ECOG			**< 0.001**			**0.002**
ECOG 1	0.950	0.627–1.438	0.808	0.670	0.420–1.069	**0.093**
ECOG 2	1.063	0.578–1.956	0.844	0.809	0.345–1.897	0.626
ECOG 3	39.862	7.050-225.383	**< 0.001**	21.352	2.918-156.271	**0.003**
Fibrinogen	1.002	1.000-1.004	**0.013**			See suppl. Table 1
Albumin	0.969	0.939-1.000	0.053			See suppl. Table 2
FAR	0.1082	1.030–1.136	**0.002**	2.058	1.197–3.537	**0.009**

Abbreviations: *NI* not included, *c* clinical staging, *p* pathological staging, *OP* operation, *HR* hazard ratio, *CI* confidence interval, *ASA* American society of anesthesiologists classification, *ECOG* Eastern Cooperative Oncology Group; Bold values indicate statistical significance

Furthermore, the ROC curve analysis was conducted to assess the predictive value of various biomarkers for OS in patients with AEG. The AUC for fibrinogen was determined to be 0.738, (*p* < 0.001; 95% CI [0.627 to 0.849]) (Fig. 2a). Conversely, albumin (Fig. 2b) exhibited an AUC of 0.378, with a *p*-value of 0.047 and a 95% CI spanning from 0.267 to 0.488. The AUC for the FAR was 0.744 (*p* < 0.001, 95% CI 0.637–0.851) (Fig. 1**)**, indicating superior predictive performance compared to both albumin and fibrinogen alone. Consequently, FAR emerges as a better predictor of OS in patients with AEG.

## Discussion

This study investigated the clinical impact of the FAR in patients who underwent primarily resection of AEG. Our findings showed that higher FAR values were significantly (*p* < 0.001) associated with lower survival rates in these patients, suggesting the potential of FAR as an independent prognostic parameter. Moreover, FAR outperformed albumin and fibrinogen in predicting patient outcomes, highlighting its enhanced predictive value for AEG patients.

The response of the immunes system inflammation, and initiation of the coagulation cascade play important roles in tumor development, with fibrinogen being primarily responsible for platelet aggregation during hemostasis. As an essential hemostatic factor, it is involved in various mechanisms that hinder antitumor immunity and therefore promoting tumor progression (Mosesson 2005). For instance, it may inhibit macrophage migration and disrupt fibrinogen-leukocyte interactions by modifying the leukocyte integrin binding motif and compromising the ability of the host to mount an effective immune response against tumors (Silva et al. 2019). Additionally, fibrinogen can inhibit the clearance of tumor cells by natural killer (NK) cells, facilitating immune evasion and metastasis (Palumbo et al. 2005). Moreover, fibrinogen has been shown to promote epithelial-mesenchymal transition (EMT) through the AKT-mTOR pathway in ESCC, contributing to tumor aggressiveness prognosis (Zhang et al. 2017). Therefore aiming a decrease of fibrinogen could be an approach for minimizing poor prognosis (Deng et al. 2023).

Similarly, there is consensus in the literature regarding the poor prognosis associated with low albumin concentrations in cancer patients (Gupta and Lis 2010), reflecting the nutritional status of patients. Malnutrition and inflammatory processes suppress albumin synthesis over time, contributing to tumor cachexia (Ballmer et al. 1994). The prognostic relevance of albumin levels in esophageal carcinomas has been well documented (Otowa et al. 2017).

While previous studies have explored the role of fibrinogen in various malignancies, including esophageal tumors, the specific prognostic value of the FAR in patients with AEG has not been investigated yet, highlighting the significance of our study. Several studies and meta-analyses have demonstrated the negative impact of high FAR values on OS in various cancers including gastrointestinal cancers (Zhang and Xiao 2019). Our study aligns with these findings, revealing significant results for several prognostic factors, including fibrinogen, albumin and FAR. Furthermore, the results of ROC curve analysis suggest that both fibrinogen and FAR outperform albumin in terms of predicting prognosis, underscoring the potential relevance of these parameters. However, further research is warranted to fully evaluate the prognostic impact of FAR in AEG and other malignancies.

The findings of our study emphasize the importance of considering FAR, especially in clinical practice for AEG patients. Patients with high preoperative FAR values may benefit from closer monitoring and tailored therapeutic interventions to address potential complications or infections.

Finally, our study highlights the underexplored prognostic significance of systemic inflammatory parameters in large western cohorts with AEG. The optimal inflammatory parameter for predicting overall survival remains uncertain (Puhr et al. 2023). However, our findings contribute valuable insights by identifying the FAR as a promising prognostic indicator for estimating OS in a sizable and representative European cohort of AEG patients.

## Limitations

The main limitation of this study was the retrospective design of our study which introduces inherent biases, notably selection bias, compounded by partial accessibility to pretherapeutic laboratory data. Additionally, being conducted solely at one center may limit the broader applicability of our findings. Furthermore, our study’s modest sample size and extended duration could impact result reliability. Furthermore, in the future it will be necessary to critically consider whether and how the FAR can be implemented into clinical routine. Dichotomous cut-offs, while necessary in clinical practice for their simplicity and ease of use, do come with limitations. They can reduce statistical power by collapsing continuous data into fixed categories, which may obscure important nuances and variability. Additionally, arbitrary or data-derived cut-offs can introduce bias and insufficiently control for confounding factors, potentially leading to misclassification. Despite these limitations, dichotomous cut-offs are often indispensable in the clinical routine, where clear thresholds are required to make quick, actionable decisions for patient care. Balancing scientific rigor with practical application remains crucial in their use.

To substantiate the clinical relevance of FAR, further investigations involving larger and more diverse patient cohorts are needed to validate this finding.

## Conclusion

In conclusion, elevated FAR values are linked to decreased OS in AEG patients undergoing primary surgery. Our study suggests that preoperative FAR serves as an independent prognostic factor in AEG patients, indicating that lower FAR levels may improve OS in this population.

## Electronic supplementary material

Below is the link to the electronic supplementary material.

Supplementary Material 1

Supplementary Material 2

Supplementary Material 3

## Data Availability

No datasets were generated or analysed during the current study.
