# Supplementary material for: Elevated fibrinogen-albumin ratio is an adverse prognostic factor for patients with primarily resected gastroesophageal adenocarcinoma
Source: J Cancer Res Clin Oncol. 2024 Oct 14;150(10):459. doi: 10.1007/s00432-024-05976-z (PMC11473574; doi:10.1007/s00432-024-05976-z)
Supplement: Supplementary file 1 — Supplementary Material 1 [file 432_2024_5976_MOESM1_ESM.docx]

| **Supplementary table 1** Multivariate Cox regression analyses estimating the influence of Fibrinogen and clinicopathologic parameters on overall survival (OS) of primary resected AEG | | | | | | |
| --- | --- | --- | --- | --- | --- | --- |
|  | Clinical staging |  |  | Pathological staging |  |  |
|  |  |  |  |  |  |  |
| ***Variable*** | HR | 95% CI | *p*-Value | HR | 95% CI | *p*-Value |
| Age65 (ref. ≥ 65) | 1.187 | 0.777-1.814 | 0.428 | 1.385 | 0.895-2.142 | 0.143 |
| SEX | 0.657 | 0.398-1.084 | 0.100 | 0.692 | 0.409-1.170 | 0.169 |
| G |  |  | **0.038** |  |  | 0.275 |
| 1 vs. 3 | 1.928 | 0.667-5.569 | 0.225 | 1.493 | 0.514-4.335 | 0.461 |
| 2 vs. 3 | 3.193 | 1.054-9.668 | **0.040** | 2.043 | 0.683-6.109 | 0.201 |
| cT |  |  | **0.006** |  |  | NI |
| 1 vs. 3 | 1.594 | 0.918-2.766 | **0.098** |  |  |  |
| 2 vs. 3 | 2.697 | 1.458-4.988 | **0.002** |  |  |  |
| cN |  |  | 0.143 |  |  | NI |
| 1 vs. 0 | 1.561 | 0.971-2.510 | 0.066 |  |  |  |
| 2 vs. 0 | 2.683 | 1.070-6.726 | **0.035** |  |  |  |
| 3 vs. 0 | 1.273 | 0.286-5.657 | 0.752 |  |  |  |
| pT |  |  | NI |  |  | **0.001** |
| 1 vs.4 |  |  |  | 3.133 | 1.629-6.024 | **<0.001** |
| 2 vs. 4 |  |  |  | 4.145 | 2.006-8.564 | **<0.001** |
| 3 vs. 4 |  |  |  | 5.171 | 1.506-17.763 | **0.009** |
| pN |  |  | NI |  |  | **0.029** |
| 1 vs. 0 |  |  |  | 2.137 | 1.215-3.757 | **0.008** |
| 2 vs. 0 |  |  |  | 2.650 | 1.216-5.775 | **0.014** |
| 3 vs. 0 |  |  |  | 2.571 | 1.085-6.091 | **0.032** |
| ASA |  |  | 0.591 |  |  | 0.234 |
| I | 0.944 | 0.483-1.845 | 0.866 | 0.501 | 0.247-1.017 | 0.056 |
| II | 0.662 | 0.248-1.769 | 0.411 | 0.396 | 0.135-1.163 | **0.092** |
| III | 0.260 | 0.028-2.448 | 0.239 | 0.426 | 0.044-4.174 | 0.464 |
| ECOG |  |  | **0.007** |  |  | **0.001** |
| ECOG 1 | 0.774 | 0.496-1.209 | 0.261 | 0.633 | 0.398-1.006 | 0.053 |
| ECOG 2 | 0.876 | 0.409-1.877 | 0.734 | 0.818 | 0.347-1.927 | 0.646 |
| ECOG 3 | 18.948 | 2.692-133.353 | **0.003** | 22.123 | 3.024-161.853 | **0.002** |
| Fibrinogen | 2.043 | 1.242-3.360 | **0.005** | 1.789 | 1.086-2.947 | **0.022** |
| Abbreviations: *NI* not included, *c* clinical staging, *p* pathological staging, *OP* operation, *HR* hazard ratio, *CI* confidence interval, *ASA* American society of anesthesiologists classification, *ECOG* Eastern Cooperative Oncology Group; Bold values indicate statistical significance | | | | | | |
|  | | | | | | |
