# Supplementary material for: Elevated fibrinogen-albumin ratio is an adverse prognostic factor for patients with primarily resected gastroesophageal adenocarcinoma
Source: J Cancer Res Clin Oncol. 2024 Oct 14;150(10):459. doi: 10.1007/s00432-024-05976-z (PMC11473574; doi:10.1007/s00432-024-05976-z)
Supplement: Supplementary file 2 — Supplementary Material 2 [file 432_2024_5976_MOESM2_ESM.docx]

| **Supplementary table 2** Multivariate Cox regression analyses estimating the influence of albumin and clinicopathologic parameters on overall survival (OS) of primary resected AEG | | | | | | |
| --- | --- | --- | --- | --- | --- | --- |
|  | Clinical staging |  |  | Pathological staging |  |  |
|  |  |  |  |  |  |  |
| ***Variable*** | HR | 95% CI | *p*-Value | HR | 95% CI | p-Value |
| Age65 (ref. ≥ 65) | 1.115 | 0.713-1.744 | 0.633 | 1.368 | 0.844-2.218 | 0.203 |
| SEX | 0.665 | 0.393-1.125 | 0.129 | 0.683 | 0.400-1.166 | 0.162 |
| G |  |  | **0.037** |  |  | 0.254 |
| 1 vs. 3 | 1.588 | 0.548-4.599 | 0.394 | 1.215 | 0.417-3.538 | 0.721 |
| 2 vs. 3 | 2.750 | 0.903-8.376 | **0.075** | 1.759 | 0.584-5.296 | 0.316 |
| cT |  |  | **0.005** |  |  | NI |
| 1 vs. 3 | 1.619 | 0.932-2.812 | 0.087 |  |  |  |
| 2. vs. 3 | 2.783 | 1.502-5.156 | **0.001** |  |  |  |
| cN |  |  | **0.039** |  |  | NI |
| 1 vs. 0 | 1.744 | 1.082-2.811 | **0.022** |  |  |  |
| 2 vs. 0 | 3.329 | 1.348-8.222 | **0.009** |  |  |  |
| 3 vs. 0 | 1.530 | 0.337-6.941 | 0.581 |  |  |  |
| pT |  |  | NI |  |  | **0.001** |
| 1 vs.4 |  |  |  | 3.167 | 1.660-6.044 | **<0.001** |
| 2 vs. 4 |  |  |  | 4.409 | 2.154-9.025 | **<0.001** |
| 3 vs. 4 |  |  |  | 5.090 | 1.455-17.804 | **0.011** |
| pN |  |  | NI |  |  | **0.009** |
| 1 vs. 0 |  |  |  | 2.219 | 1.260-3.906 | **0.006** |
| 2 vs. 0 |  |  |  | 3.081 | 1.431-6.633 | **0.004** |
| 3 vs. 0 |  |  |  | 2.960 | 1.237-7.084 | **0.015** |
| ASA |  |  | 0.703 |  |  | 0.258 |
| I | 0.977 | 0.490-1.947 | 0.948 | 0.504 | 0.247-1.027 | 0.059 |
| II | 0.727 | 0.270-1.957 | 0.528 | 0.415 | 0.140-1.233 | 0.113 |
| III | 0.303 | 0.032-2.867 | 0.298 | 0.542 | 0.055-5.360 | 0.600 |
| ECOG |  |  | **0.004** |  |  | **0.001** |
| ECOG 1 | 0.721 | 0.460-1.130 | 0.154 | 0.610 | 0.383-0.971 | **0.037** |
| ECOG 2 | 0.819 | 0.377-1.781 | 0.614 | 0.761 | 0.320-1.811 | 0.537 |
| ECOG 3 | 19.307 | 2.711-137.505 | **0.003** | 21.247 | 2.884-156.500 | **0.003** |
| Albumin | 0.808 | 0.500-1.305 | 0.384 | 0.921 | 0.568-1.493 | 0.738 |
| Abbreviations: *NI* not included, *c* clinical staging, *p* pathological staging, *OP* operation, *HR* hazard ratio, *CI* confidence interval, *ASA* American society of anesthesiologists classification, *ECOG* Eastern Cooperative Oncology Group; Bold values indicate statistical significance | | | | | | |
