# Supplementary figures and images for: Elevated fibrinogen-albumin ratio is an adverse prognostic factor for patients with primarily resected gastroesophageal adenocarcinoma
Source: J Cancer Res Clin Oncol. 2024 Oct 14;150(10):459. doi: 10.1007/s00432-024-05976-z (PMC11473574; doi:10.1007/s00432-024-05976-z)

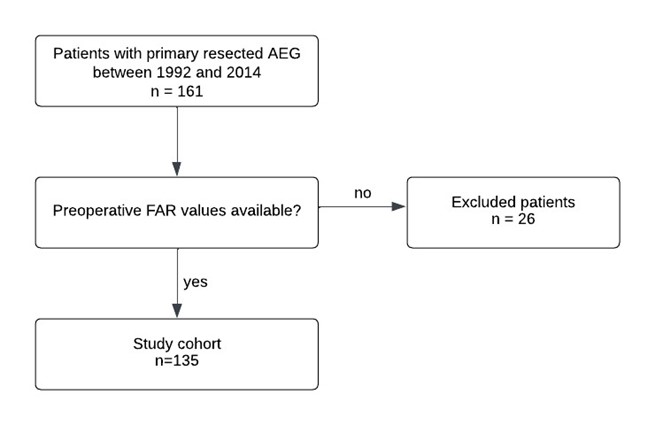

Supplement: Supplementary file 3 — Supplementary Material 3 [file 432_2024_5976_MOESM3_ESM.jpg]
